# Exploring midwives’ practice patterns and capacity for obstetric ultrasound imaging: Towards a multicentre longitudinal materno-foetal research readiness in a low-resource setting

**DOI:** 10.1371/journal.pone.0330235

**Published:** 2026-03-26

**Authors:** Albert Dayor Piersson, Philomena Ajanaba Asakeboba, Sarah Teiko Quartei, Rachel Mendy, Joseph Arkorful, Ama Boahene Akomah, Gilbertson Allorsey

**Affiliations:** 1 York St John University, School of Science, Technology & Health, Department of Diagnostic Radiography, England; 2 University for Development Studies, School of Nursing and Midwifery, Department of Advanced Nursing Practice, Tamale, Northern Region, Ghana; 3 Centre for Education, Population Health Research & Innovation, Accra, Greater Accra Region, Ghana; 4 University of the Gambia, School of Medicine and Allied Health Sciences, Department of Nursing and Reproductive Health, The Gambia; 5 University of Cape Coast, College of Health & Allied Sciences, School of Allied Health Sciences, Department of Imaging Technology & Sonography, Cape Coast, Central Region, Ghana; 6 Accra Technical University, Medical Imaging Department, Accra, Ghana; 7 Sunyani Technical University, Department of Medical Imaging and Sonography, Sunyani, Bono Region, Ghana; Kwame Nkrumah University of Science and Technology College of Health Sciences, GHANA

## Abstract

**Introduction:**

Midwives are often the first point of contact for pregnant women; yet their roles, training, and referral practices regarding obstetric ultrasound vary widely. This study aimed to explore midwives’ perspectives and experiences with obstetric ultrasound across key clinical and operational domains to assess the feasibility of conducting future multicentre maternal-foetal health research and surveillance.

**Methods:**

A descriptive cross-sectional study was conducted among 475 practicing midwives across diverse healthcare settings in Ghana. A self-administered structured questionnaire was used to collect data on midwives’ perspectives and experiences regarding obstetric ultrasound across multiple dimensions. Data analysis was performed using Microsoft Excel.

**Results:**

Most midwives were female, aged 26–35 years, held diploma qualifications, and practiced within district hospitals. Key ultrasound measures prioritised by midwives in the 1^st^ trimester include gestational age, foetal viability, estimated date of delivery (EDD), number of foetuses, and the presence of an intrauterine gestational sac. Comparatively, midwives emphasize foetal anomaly detection, amniotic fluid (liquor) volume, placental location, foetal viability, and gestational age during second trimester ultrasound screening, while in the 3^rd^ trimester screening, they prioritise foetal presentation, amniotic fluid volume, estimated foetal weight, placental location, and foetal viability. Findings suggest infrequent ultrasound reports indicating foetal anomalies. We observed a moderate perceived ability among midwives to understand foetal anomalies on obstetric ultrasound reports. Only 57.5% indicated they refer patients between one and three times for obstetric ultrasound before delivery. From the findings, it was observed that there is a predominance of sonographers undertaking obstetric ultrasound scans. Midwives may have moderate competence in interpreting obstetric ultrasound reports. An overwhelmingly positive response indicated that obstetric ultrasound improved their work performance, and a high proportion expressed interest in learning how to undertake obstetric ultrasound.

**Conclusion:**

Our findings highlight the need to standardize midwifery practices and strengthen obstetric ultrasound literacy through targeted capacity-building initiatives, not only to improve clinical decision-making but also to establish a robust foundation for scalable maternal-foetal research in low-resource settings. Additionally, our study demonstrates the potential feasibility of engaging midwives as key stakeholders in multicentre maternal-foetal research initiatives.

## Introduction

Obstetric ultrasound has become an indispensable component of antenatal care (ANC), offering critical insights into foetal development, maternal health, and pregnancy outcomes. In many healthcare settings, midwives are frontline providers of maternity services and play a crucial role in the coordination, interpretation, application of obstetric ultrasound findings, interventions and provision of appropriate treatments throughout pregnancy [1]. Their understanding of clinical indications for referral, competency in the interpretation of ultrasound reports, and recognition of foetal anomalies are vital for timely and appropriate care [2,3].

The clinical indications for obstetric ultrasound are diverse and span the entirety of prenatal care. In the first trimester, ultrasound is commonly used for confirming viability, determining gestational age, identifying multiple pregnancies, and non-identifiable images of gestational sac in utero in amenorrhoea [4]. In the second trimester, it is instrumental in anatomical surveys, interrogation of amniotic fluid volume, placenta location foetal viability, gestational age, and detection of congenital anomalies [5], while third-trimester scans are typically employed for growth monitoring and assessment of foetal wellbeing [6]. However, the extent to which midwives understand and apply these indications in practice, especially in resource-limited contexts, remains understudied.

As providers who often initiate referrals, midwives’ awareness of foetal anomalies and their frequency of observation in clinical practice can inform the urgency and justification for scans [3,7]. Moreover, patterns in how frequently patients are referred for ultrasound prior to delivery may reflect both institutional protocols and individual clinical judgment. Competence in interpreting ultrasound reports is another critical area, particularly given that many midwives rely on second-hand interpretation to inform their clinical decisions. This raises questions about the impact of ultrasound on their work performance [8] and their confidence in using ultrasound findings to guide care [9].

This study investigates midwives’ perspectives and experiences regarding obstetric ultrasound across multiple dimensions—clinical indications, key trimester-specific ultrasound measures, detection of foetal anomalies, frequency of referrals, interpretation competence, perceived work impact, and their willingness to learn ultrasound skills. The preliminary findings aim to inform not only policymaking, education, and practice, but also to support the design and feasibility of future multicentre materno-foetal health research and surveillance.

## Methods

### Study design and Setting

A cross-sectional descriptive study was conducted among practicing midwives across the regions in Ghana. The study aimed to explore referral patterns, clinical decision-making, and knowledge of obstetric ultrasound use, with an additional focus on understanding midwives’ awareness of foetal anomalies, interpretation of ultrasound reports, and their willingness to be trained in basic obstetric ultrasound. The study was designed to generate context-specific data that could inform maternal health policy and lay the foundation for multicentre materno-foetal research in Ghana.

### Participants and Sampling

We targeted 600 midwives using Cochran’s standard formula (n₀ = (z² × p × (1-p))/ e²) for survey research following this step-by-step approach:

a. Cochran’s formula (standard for surveys):
n₀ = (1.96² × 0.5 × 0.5) ÷ 0.05² = 385
(z=1.96 for 95% confidence, p = 0.5 for max variability, e = 5% margin of error)
b. Finite population correction (~12,000 total midwives):
n = 385 ÷ [1 + (385−1)/12,000] ≈ 390
c. Adjusting for 80% response rate (Ghana healthcare survey norm):
390 ÷ 0.80 ≈ 500 midwives approached and 475 responses were received.

Purposive sampling was employed to recruit midwives with diverse practice contexts (urban, peri-urban and rural regions, and both district hospitals and regional health centres), with the aim of capturing variation in experiences rather than achieving statistically representative or proportionate coverage of all midwives. Eligibility criteria included being actively involved in ANC and willing to provide informed consent.

### Data collection instrument

Data were collected using a structured, self-administered questionnaire developed by the research team based on a literature review and input from maternal health experts. The questionnaire covered the following: demographic characteristics (age, gender, years of practice, qualification, facility type, and region), clinical indications considered important for obstetric ultrasound referrals, key ultrasound parameters reviewed during first, second, and third trimester screening, frequency of patient referrals for ultrasound prior to delivery, frequency of foetal anomalies observed in practice, roles typically involved in conducting obstetric ultrasound, knowledge and interpretation of ultrasound reports, perceived impact of ultrasound on midwifery work performance, and willingness to undertake training in obstetric ultrasound. The tool was piloted among 20 midwives in a non-study region to assess clarity and relevance. Minor modifications were made based on feedback, ensuring face and content validity.

### Data analysis

Data were entered into a Google form and exported to Microsoft Excel for statistical analysis. Descriptive statistics (frequencies, percentages, means, and standard deviations) were used to summarise variables. Knowledge of interpretation of obstetrics ultrasound report and understanding of foetal anomalies on obstetric ultrasound report were grouped into three score categories: 1–3, 4–6, and 7–10; while referral frequencies were grouped into three categories: 1–3 times, 4–6 times, and 7–10 times (Table 1). The mean, mode, and range of referral frequencies were calculated to describe common patterns of ultrasound use. For items with multiple-response options (e.g., ultrasound roles or clinical indications), response frequencies were calculated. Frequency distributions were visualised using pie and bar charts in Excel to illustrate distribution patterns and key findings.

**Table 1:** Interpretation of knowledge of interpretation of obstetrics ultrasound report, understanding of foetal anomalies on obstetric ultrasound report, and referral frequencies.

knowledge of interpretation of obstetrics ultrasound report and understanding of foetal anomalies on obstetric ultrasound report		Referral Frequencies	
Score Range	Interpretation	Referral Frequency Group	Range
Low knowledge	1 - 3	Low	1-3 times
Moderate knowledge	4 - 6	Moderate	4-6 times
High knowledge	7 - 10	High	7-10 times

**Legend:** Knowledge of interpretation of obstetric ultrasound reports and understanding of foetal anomalies were assessed using a composite score. Knowledge levels were categorised as low (scores 1–3), moderate (scores 4–6), and high (scores 7–10). Referral frequency was similarly grouped as low (1–3 referrals), moderate (4–6 referrals), and high (7–10 referrals) within the specified ranges.

### Ethical considerations

Ethical approval for this study was obtained from the Centre for Education, Population Health Research & Innovation (Approval Number: CEPHRI/erc/2025/0781). Midwife WhatsApp group administrators across various regions were contacted and granted approval to disseminate a self-administered questionnaire, designed using Google Forms, via their platforms. To ensure broader participation—especially in areas with limited internet access—hard copies of the questionnaire were also distributed. Additionally, faculty members who engaged with midwives during seminars and workshops facilitated further dissemination across regions. All participants were provided with detailed information about the study objectives, procedures, potential risks, and benefits, and were given the opportunity to ask questions before participation. Informed written consent was obtained from all participants before enrolment, with consent documented through signed consent forms in accordance with the approved ethical protocol and the principles of the Declaration of Helsinki. To maintain confidentiality, data were anonymised and used exclusively for research purposes.

## Results

**Fig 1:**
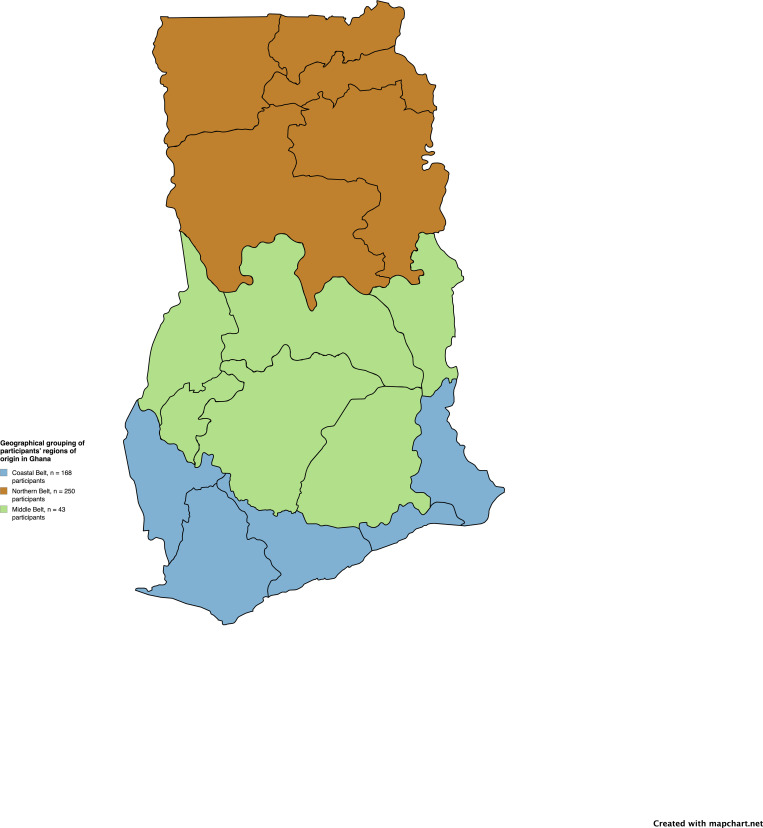
Geographical grouping of participants’ regions of origin in Ghana. Regions are clustered into Northern (Northern, Upper East, Upper West, North East, and Savannah regions), Middle (Ashanti, Bono, Bono East, Ahafo, Eastern, and Oti regions), and Coastal belts (Greater Accra, Central, Western, Western North, and Volta regions) to aid interpretation for readers unfamiliar with Ghana’s social geography (No response = 14). Map was generated from https://www.mapchart.net/africa-detailed.html.

The demographic profile (Table 2) of the study participants revealed that the majority of midwives were within the 26–35 years age range, with over 95% identifying as female. Most respondents had between 0–5 years of clinical experience, held a Diploma qualification in midwifery, and were predominantly employed in district-level hospitals. A significant proportion of the participants were based in the Northern belt, reflecting the geographical distribution of midwifery practice within the study context (see Fig 1).

**Table 2:** Demographics.

Items	Midwives, n = 475
Age (years)	
18-25	67 (14.4)
26-30	145 (31.3)
31-35	135 (29.1)
36-40	89 (19.2)
≥ 41	30 (6.5)
No response	9
Sex	
Male	19 (4.1)
Female	440 (95.9)
No response	14
No of years in midwifery practice	
0-5	275 (59.1)
6-10	159 (34.2)
11-15	22 (4.7)
16-20	7 (1.5)
≥ 21	2 (0.4)
No response	7
Current midwifery qualification	
Certificate	45 (9.7)
Diploma	351 (76.0)
BSc	63 (13.6)
Postgraduate	2 (0.4)
Other	4 (0.9)
No response	11
Type of facility of work	
CHPS	29 (6.3)
Health centre	82 (17.9)
Municipal Hospital	85 (18.5)
District Hospital	160 (34.9)
Regional Hospital	22 (4.8)
Teaching Hospital	40 (8.7)
Private Hospital	34 (7.4)
CHAG	4 (0.9)
Others	5 (1.1)
No response	14

**Legend:** Data are presented as frequency *n* and percentage (%), unless otherwise stated. Percentages are calculated based on the total number of respondents (*n* = 475) and may not sum to 100% due to rounding or missing responses. “No response” indicates missing data. CHPS = Community-based Health Planning and Services; CHAG = Christian Health Association of Ghana.

## Discussion

This study reveals midwives’ prioritisation patterns for obstetric ultrasound indications and measures across trimesters, alongside their self-reported knowledge of anomaly recognition, referral practices, and task-shifting willingness. Key findings highlight appropriate clinical focus on high-risk conditions (vaginal bleeding, uterine anomalies, hydatidiform mole) and core parameters (gestational age, foetal viability, presentation), but also reveal specific gaps in biometry familiarity and standardised interpretation. The key findings are discussed below.

### Clinical indications for obstetric ultrasound

**Fig 2:**
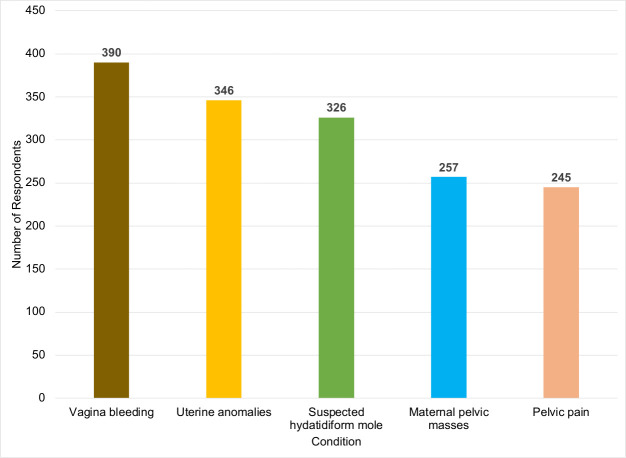
Clinical indications considered important for obstetric ultrasound.

When asked about indications beyond routine antenatal scans, midwives most often identified vaginal bleeding, uterine anomalies, and suspected hydatidiform mole, an abnormal placental proliferation visible sonographically (Fig 2). This pattern reflects an emphasis on high-risk maternal conditions in a context where late presentation to ANC and limited specialist access remain common in LMICs and where medically complicated pregnancies are frequent [10,11]. By prioritising these indications, midwives are using ultrasound as a targeted tool for timely risk stratification and maternal–foetal surveillance rather than as a purely routine screening test.

### Key ultrasound measures considered by midwives

**Fig 3:**
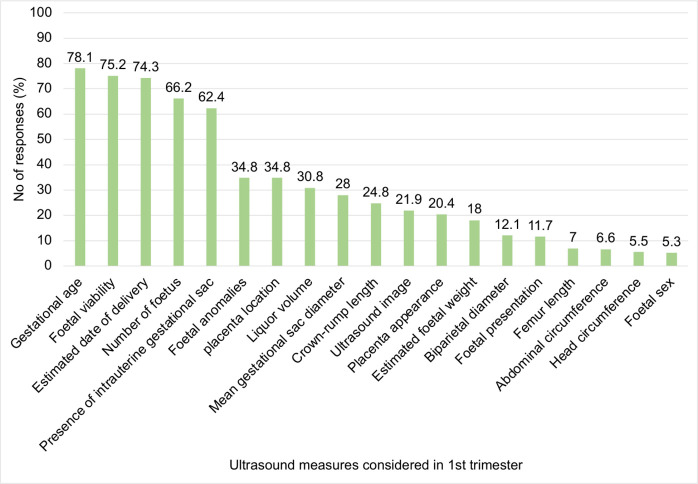
Key Ultrasound Measures Considered by Midwives During 1st Trimester Prenatal Screening.

In the first trimester, midwives prioritised gestational age, foetal viability, estimated date of delivery, number of foetuses, and presence of an intrauterine gestational sac (**Fig 3**), which is consistent with recommended core components of early pregnancy assessment [4,12]. These measures are central for accurate dating, early identification of multiple gestation, and detection of early pregnancy loss or ectopic gestation, all of which strongly influence maternal and perinatal outcomes in LMICs. Notably, crown–rump length—which provides a more precise estimate of gestational age than gestational sac diameter between 10 and 14 weeks’ gestation [13,14] was accorded relatively low importance, suggesting a specific skills or knowledge gap in optimal first-trimester biometry.

In the late first trimester, ultrasound can also facilitate early detection of major structural abnormalities and, where available, first-trimester aneuploidy screening through nuchal translucency (NT) measurement [4,15–17]. Although NT was not included in our items, its omission from routine practice in this setting is likely and should be explored in future work, given its increasing integration into early pregnancy care in higher-income settings.

**Fig 4:**
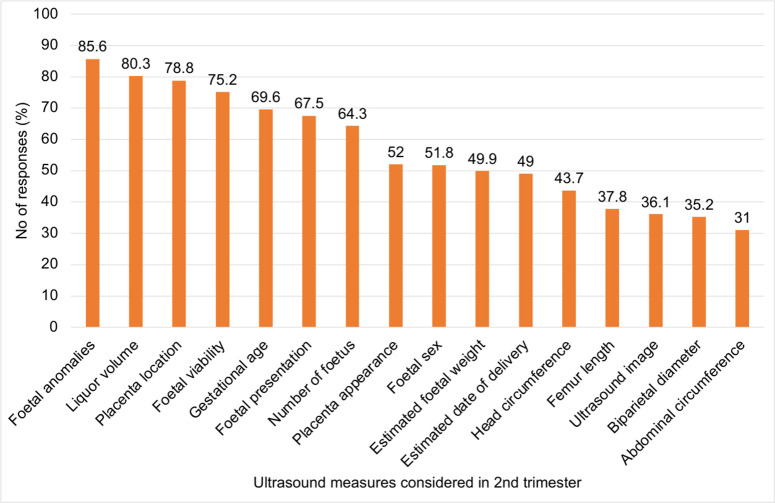
Key Ultrasound Measures Considered by Midwives During 2nd Prenatal Screening.

During the second trimester, midwives emphasised foetal anomaly detection, amniotic fluid volume, placental location, foetal viability, and gestational age (**Fig 4**), aligning with global recommendations for mid-pregnancy scanning. However, relatively low priority was given to biparietal diameter, head circumference, abdominal circumference, and femur length, despite their central role in estimating foetal size and weight and in providing alternative dating parameters when no earlier scan is available. This pattern points to an opportunity for targeted training in basic biometry and its interpretation, particularly in LMIC contexts where accurate assessment of these parameters can support earlier detection of growth disorders, placental dysfunction, and other complications.

**Fig 5:**
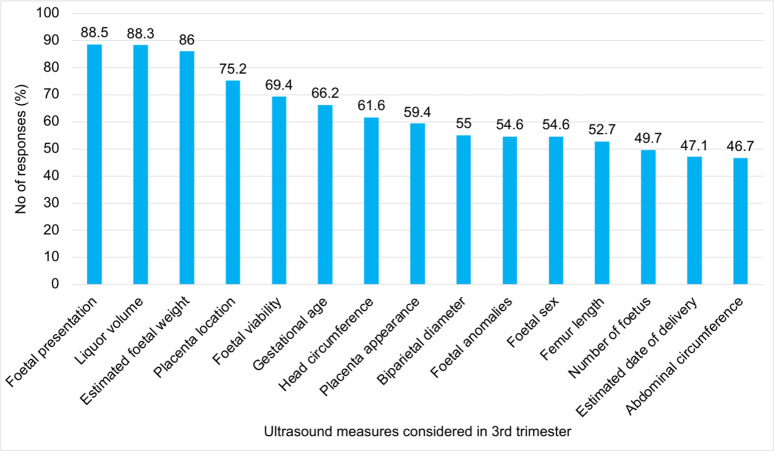
Key Ultrasound Measures Considered by Midwives During 3rdTrimester Prenatal Screening.

In the third trimester, midwives prioritised foetal presentation, amniotic fluid volume, estimated foetal weight, placental location, and foetal viability (Fig 5), which are key to intrapartum planning and prevention of obstructed labour, foetal distress, and stillbirth. This aligns with international guidelines [6,12], Third-trimester ultrasound can also support late gestational age assessment and foetoplacental Doppler evaluation where earlier scans are unavailable [6], underscoring the need for context-appropriate protocols that guide midwives on when and how to request such assessments.

### Frequency of Foetal Anomalies Observed in Practice and Awareness of Foetal Anomalies

**Fig 6:**
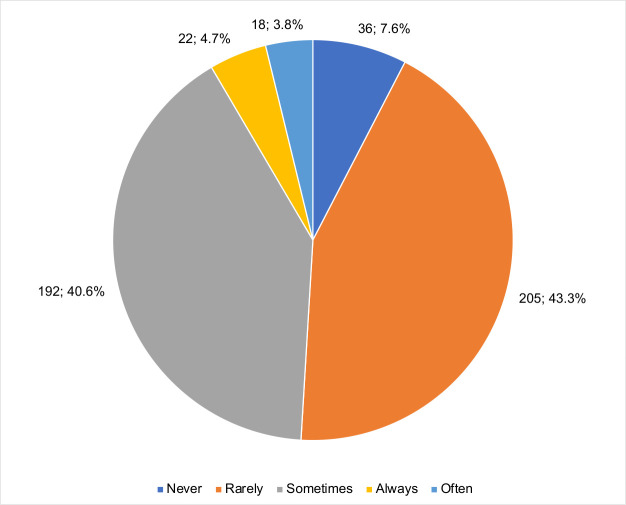
Frequency of foetal anomalies observed in practice.

Most midwives reported that ultrasound findings of foetal anomalies were “rarely” or “sometimes” encountered (Fig 6), a pattern that may reflect true low prevalence, but also limited detection or under-reporting. In contrast, hospital-based data from Ghana demonstrate a substantial burden of congenital anomalies detected on prenatal ultrasound, including ventriculomegaly, neural tube defects, abdominal wall defects, and orofacial anomalies [18]. This discrepancy suggests that many anomalies may not be identified or recognised in lower-level facilities, possibly due to restricted scan protocols, limited anomaly-focused training, or documentation practices.

Midwives in our study reported a moderate perceived ability to understand foetal anomalies described in ultrasound reports (Table 3), echoing earlier work in Ghana indicating challenges in recognising neonates with congenital anomalies [3]. Limited knowledge of congenital anomalies was also documented among the general public and pregnant women [19–21], while foetal anomaly diagnoses are associated with substantial psychological burden for parents. In LMIC settings, where access to specialised foetal medicine and continuous professional development is constrained, strengthening midwives’ competencies in recognising, interpreting, and communicating anomaly findings is likely to improve referral pathways and enhance the quality of multicentre maternal–foetal research

**Table 3:** Knowledge of obstetric ultrasound report interpretation, awareness of foetal anomalies, and frequency of patient referrals prior to delivery.

Score Range	Knowledge of interpretation of obstetrics ultrasound report		Understanding of foetal anomalies on obstetric ultrasound report		Frequency of patient referrals for obstetric ultrasound prior to delivery		
	n (%)	Mean ± SD	n (%)	Mean ± SD	n (%)	Weighted Average	Mode
1–3	111 (23.5)	5.19 ± 2.25	127 (26.9)	5.11 ± 2.26	272 (57.1)	4.44	3
4–6	219 (46.3)		253 (53.5)		106 (22.4)		
7–10	143 (30.2%)		93 (19.7)		95 (20.1)		

**Legend:** This table summarises respondents’ self-reported knowledge and practices related to obstetric ultrasound reports. Scores were categorised into three ranges (1–3 = low, 4–6 = moderate, 7–10 = high) and presented as frequencies and percentages [n (%)]. Mean ± standard deviation (SD) describes central tendency for knowledge-related domains, while weighted average and mode summarise the frequency of patient referrals for obstetric ultrasound prior to delivery.

### Frequency of patient referrals prior to delivery

Over half of the midwives reported referring women for obstetric ultrasound between one and three times prior to delivery (Table 3), exceeding the minimum recommendation of at least one scan before 24 weeks’ gestation and signalling a growing integration of ultrasound into ANC. The mean referral frequency of four scans is similar to previous observations of 3–4 scans per pregnancy in other settings [22], but the wide variation in referral frequency points to heterogeneous practice rather than a consistently applied protocol. This variability likely arises from interacting factors, including differences in clinical experience, facility resources, local guidelines, and patient-level determinants such as late ANC initiation, travel distance, socioeconomic constraints, and cultural expectations. Other studies have also highlighted some of these factors [23,24]. Ghana’s Free Maternal Healthcare Policy under the National Health Insurance Scheme may have facilitated greater access, while incomplete removal of out-of-pocket costs and uneven service distribution continue to shape utilisation [25–27]. The finding that nearly 43% of midwives refer women four or more times raises questions about potential overuse for reassurance, medico-legal concerns, financial incentives in some facilities, and gaps in standardised referral criteria. For future multicentre trials, these patterns underscore the need to plan budgets assuming a substantial proportion of participants may undergo frequent scanning. This is consistent with WHO emphasis on increased ANC contacts to improve outcomes and satisfaction [28].

### Frequency of Roles Performing Obstetric Ultrasound

**Fig 7:**
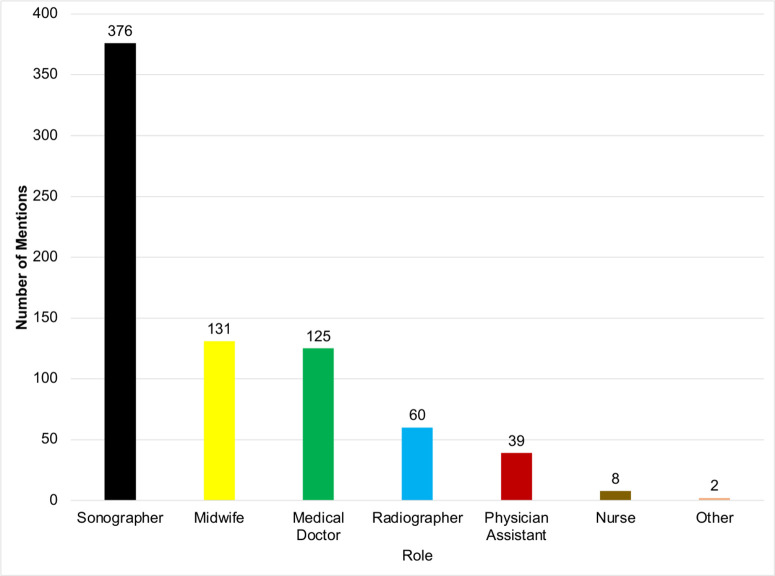
Frequency of roles performing obstetric ultrasound.

Sonographers were the most frequent providers of obstetric ultrasound (Fig 7**),** reflecting recent national efforts to expand diagnostic sonography training at tertiary institutions and address workforce shortages. For multicentre maternal–foetal research, however, there will be a need to incorporate highly specialised sonographers with advanced competencies in areas such as neurosonography and foetal echocardiography, alongside robust quality assurance systems [29,30]. Short ultrasound training courses for physicians [31,32] and midwives [3] have demonstrated positive effects on local practice, yet concerns persist regarding unregulated practice, variable training quality, and the proliferation of unlicensed providers in Ghana [33]. While task-shifting ultrasound to midwives and other health professionals is feasible and consistent with global guidance [12], it must be accompanied by clearly defined scopes of practice, competency-based curricula, supervision, and mechanisms to minimise interprofessional variability in scan quality and report interpretation. These considerations are particularly important when designing standardised protocols for multicentre research in LMICs.

### Knowledge of Obstetric Ultrasound Report Interpretation

Our findings also indicate midwives may have a moderate competence in interpreting obstetric ultrasound reports (Table 3), highlighting potential variability in training and confidence levels across clinical settings. This compares with studies in other African countries that have reported a high performance in carrying out obstetrics ultrasound [34,35], suggesting a corresponding high knowledge of the interpretation. However, the moderate knowledge evidenced from our study may reflect the lack or limited structured ultrasound education in midwifery curricula, posing a barrier to optimal utilization of ultrasound findings in clinical decision-making. There is the indication that the training of midwives on obstetric ultrasound scans in some African countries remains a serious challenge; however, there is a need to incorporate obstetric ultrasound scans as part of the scope of practice of midwives [1]. For instance, opportunities abound to adopt training curricula on basic scans that is inclusive of didactic and supervised clinical practicum components [36]. Addressing these knowledge gaps is critical not only for improving clinical outcomes but also for ensuring the reliability of data collection in prospective multicentre maternal–foetal research initiatives in LMICs.

Midwives’ self-reported knowledge of obstetric ultrasound report interpretation was moderate (Table 3), suggesting uneven training and confidence across facilities. Studies from other African contexts report that, when adequately trained, midwives and other non-physician clinicians can perform obstetric ultrasound to a high standard [34,35]. The contrast with our findings suggests that structured ultrasound education is currently limited in midwifery programmes and in-service training in Ghana, constraining the full integration of ultrasound findings into clinical decision-making. Incorporating basic obstetric ultrasound—covering essential physics, image acquisition, standard measurements, and report interpretation—into midwifery curricula and supervised clinical practice would be helpful.

### Perceived Impact of Ultrasound on Midwifery Work Performance and Willingness to Learn How to Undertake Obstetric Ultrasound

**Fig 8:**
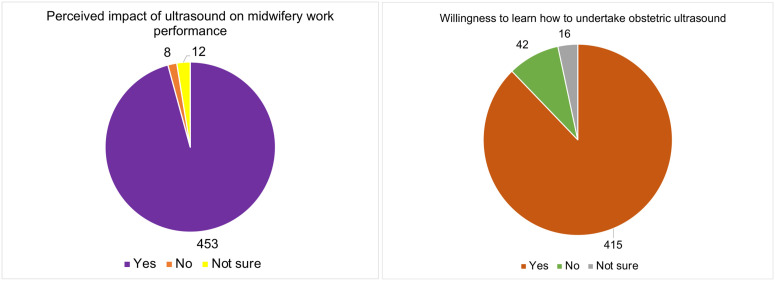
Perceived impact of ultrasound on midwifery work performance and willingness to learn how to undertake obstetric ultrasound.

Most midwives reported that access to obstetric ultrasound improves their work performance (Fig 8), consistent with evidence that ultrasound can strengthen confidence in gestational dating, enhance risk assessment, and support timely referral in African settings [8,37]. This perceived benefit, however, is contingent on reliable equipment, clear protocols, and adequate training; without these, reliance on ultrasound may paradoxically delay decisions or generate uncertainty.

A large proportion of midwives also expressed willingness to learn how to perform obstetric ultrasound themselves (Fig 8), indicating substantial latent capacity for task-sharing in contexts where sonographers are scarce. At the same time, some studies report midwives’ reservations about assuming scanning responsibilities due to safety concerns, workload, and traditional role boundaries [2,37]. Given persistent difficulties in recruiting and retaining sonographers particularly in rural and underserved areas, a combined strategy that expands formal sonography training while providing carefully structured, competency-based basic ultrasound training and ongoing refresher programmes for midwives may be the most pragmatic approach. Such a strategy aligns with WHO guidance on ANC contacts [28] and could help ensure that women in remote settings benefit from timely, high-quality ultrasound while safeguarding image quality, diagnostic accuracy, and patient safety.

### Strengths and Limitations

Our study is one of the few to systematically explore obstetric ultrasound practices from the perspective of midwives in LMIC settings, offering rich, context-specific insights relevant for maternal-foetal health system strengthening. Its large, multi-facility sample enhances the generalisability of findings across diverse clinical environments.

There are also limitations worth highlighting in our study. A key limitation of this study lies in its reliance on self-reported data, which may be subject to recall bias, social desirability bias, and variability in practice. In addition, we did not compare responses between facilities, and we did not independently verify clinical practice or diagnostic accuracy. Further, we focused solely on midwives. Finally, our study did not consider several other areas of midwifery practices.

### Conclusion and Implication for Health Policy and Prospective Multicentre Research Practices

Our findings suggest that midwives in this setting already use obstetric ultrasound in ways that are broadly aligned with international guidance, while revealing important gaps in biometry, anomaly recognition, and report interpretation that have implications for both routine care and research. Strengthening structured, competency-based ultrasound education for midwives and other frontline providers, supported by clear national guidelines, quality assurance systems, and regulated task-sharing, could enhance the safety and effectiveness of ultrasound-enabled ANC in LMICs.

For future multicentre studies, it will be important to characterise sonographers’ practices, understand pregnant women’s experiences of ultrasound and communication around findings, and evaluate strategies that promote shared decision-making, including enabling women and partners to view the screen and discuss results in real time. Research should also examine how well current practices communicate the limitations of ultrasound, particularly regarding prediction of birthweight, mode of delivery, and detection of certain anomalies in early pregnancy, to ensure that expanding access to ultrasound is accompanied by realistic expectations and informed, respectful care.
